# Contribution of Short Chain Fatty Acids to the Growth of *Pseudomonas aeruginosa* in Rhinosinusitis

**DOI:** 10.3389/fcimb.2020.00412

**Published:** 2020-08-11

**Authors:** Do-Yeon Cho, Daniel Skinner, Ryan C. Hunter, Christopher Weeks, Dong Jin Lim, Harrison Thompson, Christopher R. Walz, Shaoyan Zhang, Jessica W. Grayson, William E. Swords, Steven M. Rowe, Bradford A. Woodworth

**Affiliations:** ^1^Department of Otolaryngology Head and Neck Surgery, University of Alabama at Birmingham, Birmingham, AL, United States; ^2^Gregory Fleming James Cystic Fibrosis Research Center, University of Alabama at Birmingham, Birmingham, AL, United States; ^3^Division of Otolaryngology, Department of Surgery, Veteran Affairs Medical Center, Birmingham, AL, United States; ^4^Department of Microbiology and Immunology, University of Minnesota, Minneapolis, MN, United States; ^5^Department of Medicine and Microbiology, University of Alabama at Birmingham, Birmingham, AL, United States; ^6^Department of Medicine, Pediatrics, Cell Developmental and Integrative Biology, University of Alabama at Birmingham, Birmingham, AL, United States

**Keywords:** anaerobes, short chain fatty acid, pseudomonas, mucin-fermenter, fermentation, rhinosinusitis, Chronic sinusitis, hypoxia

## Abstract

**Background:** Chronic rhinosinusitis (CRS) is characterized by complex bacterial infections with persistent inflammation. Based on our rabbit model of sinusitis, blockage of sinus ostia generated a shift in microbiota to a predominance of mucin degrading microbes (MDM) with acute inflammation at 2 weeks. This was followed by conversion to chronic sinus inflammation at 3 months with a robust increase in pathogenic bacteria (e.g., *Pseudomonas*). MDMs are known to produce acid metabolites [short chain fatty acids (SCFA)] that have the potential to stimulate pathogen growth by offering a carbon source to non-fermenting sinus pathogens (e.g., *Pseudomonas*). The objective of this study is to evaluate the concentrations of SCFA within the mucus and its contribution to the growth of *P. aeruginosa*.

**Methods:** Healthy and sinusitis mucus from the rabbit model were collected and co-cultured with the PAO1 strain of *P. aeruginosa* for 72 h and colony forming units (CFUs) were determined with the targeted quantification of three SCFAs (acetate, propionate, butyrate). Quantification of SCFAs in healthy and sinusitis mucus from patients with *P. aeruginosa* was also performed via high performance liquid chromatography.

**Results:** To provide evidence of fermentative activity, SCFAs were quantified within the mucus samples from rabbits with and without sinusitis. Acetate concentrations were significantly greater in sinusitis mucus compared to controls (4.13 ± 0.53 vs. 1.94 ± 0.44 mM, *p* < 0.01). After 72 h of co-culturing mucus samples with PAO1 in the presence of mucin medium, the blue-green pigment characteristic of *Pseudomonas* was observed throughout tubes containing sinusitis mucus. CFUs were higher in cultures containing mucus samples from sinusitis (8.4 × 10^9^ ± 4.8 × 10^7^) compared to control (1.4 × 10^9^ ± 2.0 × 10^7^) or no mucus (1.5 × 10^9^ ± 2.1 × 10^7^) (*p* < 0.0001). To provide evidence of fermentative activity in human CRS with *P. aeruginosa*, the presence of SCFAs in human mucus was analyzed and all SCFAs were significantly higher in CRS with *P. aeruginosa* compared to controls (*p* < 0.05).

**Conclusion:** Given that SCFAs are solely derived from bacterial fermentation, our evidence suggests a critical role for mucin-degrading bacteria in generating carbon-source nutrients for pathogens. MDM may contribute to the development of recalcitrant CRS by degrading mucins, thus providing nutrients for potential pathogens like *P. aeruginosa*.

## Introduction

Chronic rhinosinusitis (CRS), characterized by dysfunctional mucociliary clearance (MCC) with subsequent microbial colonization, is known as a multifactorial disease process where bacterial infection may play a role in the commencement or progression of the inflammatory response (Ramakrishnan et al., 2013). CRS patients have complex sinus microbial communities that incite persistent inflammation and airway damage (Lee and Lane, 2011). Despite the high density of bacteria that colonize the airway, nutrient sources that sustain bacterial growth *in vivo* and the derivation of those nutrients are not well-characterized. Recently, our laboratory successfully created a rabbit model of CRS by blocking the sinus drainage pathway (Cho et al., 2017a). Key data generated from this model indicate a clear sequence of events that augment our understanding of recalcitrant CRS pathophysiology: (1) blockage of sinus ostia generates a shift in microbiota to a predominance of anaerobes and (2) the shift from acute to chronic sinus inflammation is subsequently associated with a robust increase in pathogenic bacteria (e.g., *Pseudomonas*). How the two events are mechanistically related is unknown, but critical to understanding disease pathogenesis and the potential for intervention.

Abundant nutrient sources are provided by airway mucus to living organisms in the microenvironment and mucins are the major macromolecular constituents of mucus that provide a large carbon reservoir [e.g., short chain fatty acids (SCFA)] (Flynn et al., 2016). Mucins are the main nutrient source for niche-specific microbiota of the gut and oral cavity (Flynn et al., 2016). Therefore, mucin degrading microbes (MDM, primary mucin degrader) are thought to modify the nutritional landscape of the microenvironment and stimulate the growth of secondary colonizers (Kolenbrander, 2011). It is well-known that similar interactions exist between the commensal gut microbiota and the mucus layer of the human intestine (Cameron and Sperandio, 2015). Although the common sinus pathogen, *Pseudomonas aeruginosa*, cannot derive SCFAs endogenously (Flynn et al., 2016), little is known regarding the degradation of airway mucins as a source of SCFAs by these or other opportunistic pathogens in the upper airway.

Data from observational studies (Tunney et al., 2008) and the rabbit model (Cho et al., 2017a) indicate a shift in the microbiota to predominately anaerobic bacteria with impaired MCC. In rabbits, production of bioavailable nutrients (SCFA) for pathogenic bacteria follows and there was a subsequent shift from acute to chronic inflammation with robust generation of pathogenic microbes (e.g., *P. aeruginosa*). Since *P. aeruginosa* cannot derive SCFAs (e.g., acetate and propionate) from the host mucus through fermentation on their own, we hypothesized that anaerobic bacteria may ferment mucins into SCFA forms usable by *P aeruginosa*. This would provide a novel mechanistic basis for recalcitrant CRS pathogenesis following MCC disruption that occurs with acute respiratory infections/inflammation and subsequent compromise of the sinus ostia by edema (Crabbe et al., 2014). Therefore, the objective of this study is to evaluate the concentrations of SCFA within the sinonasal mucus (from rabbit and human) and its contribution to the growth of *P. aeruginosa*.

## Methods

### PAO1 Stock Preparation

*Pseudomonas aeruginosa* (PAO1 strain) was expanded from glycerol frozen stock by inoculating 50 ml of lysogeny broth (LB) followed by overnight growth at 37°C on a shaker at 200 rpm. Cultures were streaked on LB agar plates according to the quadrant method and grown in a static incubator at 37°C overnight at least twice to confirm conformity of cultures. From the plate, an isolated colony was grown in 10 ml of LB-Miller broth at 37°C on a shaker at 200 rpm overnight. Cultures were diluted with fresh LB-Miller broth to an inoculation concentration of 1 × 10^4^ to make a PAO1 stock for further experiments.

### Animal Model

This study was approved by the Institutional Animal Care and Use Committee (IACUC Approval number 21687) at the University of Alabama at Birmingham (UAB). *Pasteurella*-free, female, New Zealand white rabbits (2–4 kg) were used for the study. Before initiation, rabbits were acclimatized to the animal facility for at least 1 week. For any procedure, rabbits were anesthetized with [ketamine (20 mg/kg) (MWI, Boise, ID), dextomitor (0.25 mg/kg) (Zoetis Inc., Kalamazoo, MI), buprenorphine (0.03 mg/kg) (Reckitt Benckiser Pharmaceuticals Inc., Richmond, VA), and carprofen (5 mg/kg) (Zoetis Inc., Kalamazoo, MI)] in a warm room for comfort. Rabbits did not receive any antibiotics before or during this study.

### Mucus Collection From Rabbit Model of Sinusitis

Based on our previous experiments, MDMs dominated on week 2 after blocking the sinus drainage pathway in the rabbit and therefore mucus samples were collected at week 2 (Cho et al., 2017a). A total of four rabbits were used to create a rabbit model of acute sinusitis without providing exogenous bacteria or pathogens as described previously (Cho et al., 2017a). A sterile synthetic sponge (Merocel®, Medtronic, Minneapolis, MN) was inserted in the left (unilateral) middle meatus (natural outflow tract of maxillary sinus) of New Zealand white rabbits for 2 weeks and the sponge was removed on week 2. To confirm acute sinusitis, rabbits were examined with micro-computed tomography (microCT) scanning using SPECT/CT (X-SPECT system, Gamma Medica, Northridge, CA) and nasal endoscopy [1.7 mm 30-degree endoscope (Karl Storz, Tuttlingen, Germany)] on Week 0 and 2. Mucus samples were collected on Week 0 (control) and Week 2 (sinusitis) using a special suction catheter created by our laboratory. To evaluate whether targeting fermentative anaerobes halts the sinusitis progression from acute to chronic, metronidazole (50 mg/kg, twice a day for 5 days) was administered to the acute sinusitis rabbits (week 2). MicroCT scans were repeated at week 6 (between acute and chronic) to assess for CT evidence of sinusitis (opacification). Sinus opacification grading was performed using Kerschner's rabbit Sinus CT grading system [scoring each imaging study based on estimated percent (%) opacification of the maxillary sinus: 1 for <10%, 2 for 10–19%, 3 for 20–29%, 4 for 30–39%, 5 for 40–49%, 6 for 50–59%, 7 for 60–69%, 8 for 70–79%, 9 for 80–89%, and 10 for 90% ≤] (Kerschner et al., 2000). Opacification % was measured using the ImageJ version 1.50i (National Institutes of Health, Bethesda, MD) by two blinded judges.

### *In vitro* Co-culturing

To test whether MDMs at week 2 are able to generate metabolites from mucin that could simultaneously stimulate *P. aeruginosa* growth, mucus samples collected at week 2 were co-cultured with PAO1. In a polystyrene culture tube (Fisher Scientific Company, Pittsburg PA), a bottom agar plug was made by adding 900 μL of 0.7% agar inoculated with 100 μL of mucus collected from rabbits (Day 0 or Week 2) or 0.7% agar (negative control) (*n* = 4). After allowing this to solidify, a top plug was made with 450 μL of 1.5% minimal media agar inoculated with the 1/1,000 dilution of an overnight culture of *P. aeruginosa* (PAO1). After solidification, co-cultures were placed at 37°C for 72 h. Agar plugs were then removed from the upper phase and homogenized by pipetting in 10 mL of phosphate buffered saline. Colony forming units (CFU) per tube were determined by serial dilution and plating on LB agar.

### Mucus Collection and Culture From Human Chronic Sinusitis

To understand the concentration of SCFAs in human CRS with *P. aeruginosa*, mucus samples were collected from the middle meatus. This study was approved by the Institutional Review Board (IRB approval number 300003118) at the University of Alabama at Birmingham and all patients provided written informed consent. Subjects (≥18 years of age) visiting the University of Alabama at Birmingham sinus clinic were recruited for the study. Patient eligibility criteria were designed to limit enrollment to healthy individuals and patients who clearly have CRS based on Sinus and Allergy health partnership criteria (Benninger et al., 2003; Orlandi et al., 2016). Control patients were enrolled based on endoscopic procedures for unilateral benign tumors (where the opposite side could be tested) and other disease entities where sinus inflammation was not present (e.g., CSF leaks, nasal septal deviation, benign nasal tumor, and turbinate hypertrophy). Demographic and clinical data were prospectively collected, de-identified, and stored in a securely encrypted electronic database. For cultures, specimens were obtained in the clinic or operating room (OR) with ESwabs (COPAN Diagnostics, Inc., Murrieta, CA) for hospital laboratory culture. Culture swabs were endoscopically guided to the area of interest with care taken to avoid contamination from the nares. The mucosal surface and overlying mucus of the middle meatus (from frontal, ethmoid, and/or maxillary sinuses) was aggressively swabbed for at least five full rotations until fully saturated. Culture swabs were sent to the hospital clinical microbiology laboratory for aerobic and anaerobic culture for bacterial growth and isolation. For metabolite quantification, mucus samples from the area of interest were collected in the clinic or OR using a modified catheter suction created by our laboratory (Cho et al., 2017a,b; Durmowicz et al., 2018).

### Metabolite Quantification

Targeted quantification of SCFAs was performed via high performance liquid chromatography (HPLC). The system consisted of a Shimadzu SCL-10A system controller, LC-10AT liquid chromatograph, SIL-10AF autoinjector, SPD-10A UV-Vis detector, and CTO-10A column oven. Separation of compounds was performed with an Aminex HPX-87H guard column and an HPX-87H cation-exchange column (Bio-Rad, Hercules, CA). The mobile phase consisted of 0.05 N H_2_SO_4_, set at a flow rate of 0.5 mL min^−1^. The column was maintained at 50°C and the injection volume was 50 μL. Amino acid and metabolite (acetate and propionate) quantification from enrichment supernatants were performed by Millis Scientific, Inc. (Baltimore, MD) using liquid chromatography–mass spectrometry and gas chromatography–mass spectrometry (GC–MS). Samples for amino acid quantification were spiked with 1 μL of uniformly labeled amino acids (Cambridge Isotope Labs) and derivatized using AccQ-Tag reagent (Waters Corp.) for 10 min at 50°C. A Waters Micromass Quatro LC–MS interfaced with a Waters Atlantis dC18 (3 μm 2.1 × 100 mm) column was used. Reverse-phase LC was used for separation (mobile phases A: 10 mM ammonium formate in 0.5% formic acid, B: methanol) with a constant flow rate (0.2 mL min^−1^) and a column temperature of 40°C. Electrospray ionization was used to generate ions in the positive mode and multiple reaction monitoring was used to quantify amino acids. Samples (~100 μL) for acetate and propionate quantification were first diluted (150 μL water), spiked with internal standards (10 μL of 1,000 ppm acetate [^13^C_2_] and 1,000 ppm propionate [^13^C_1_]) and acidified using 2 μL of 12N HCl. After equilibration at 60°C for 2 h, carboxen/polydimethylsiloxane solid phase microextraction (SPME) fiber was used to adsorb the headspace at 60°C for 30 min. Acids were then desorbed into the gas chromatograph inlet for 2 min. A 30 m × 0.32 mm ID DB-624 column attached to a Thermo Electron Trace gas chromatograph with helium carrier gas (2.0 mL min^−1^) was used for separation of analytes. A Waters Micromass Quatro GC mass spectrometer was used for detection and quantification of target ions.

### Effect of SCFA on PAO1 Growth

To test the effects of SCFA on *P. aeruginosa* growth, we incubated PAO1 strains with SCFA at varying concentrations. Each SCFA medium was prepared by adding individual SCFA to M9 minimal salts media at different concentrations, obtained from Sigma (St. Louis, MO). PAO1 seeding solution was prepared by adding 300 μl PAO-1 stock to 50 ml LB-Miller broth. 100 μl of PAO1 seeding solution was inoculated into 700 μl SCFAs media solutions on a 48-well plate and incubated at 37°C for 12 h. The final concentration of each SCFA was 0.01, 0.1, 1, or 10 mM based on *in vivo* HPLC data. Optical density (OD) of planktonic PAO1 growth was measured at 600 nm using a microplate reader. Additionally, to assess the growth of PAO1 strains in the presence of all 3 SCFAs, the colony counts of PAO1 were compared between the single (the most dominant SCFA) vs. 3 SCFAs after incubating 12 h in M9 minimal salts media (repeated × 10).

### Statistical Analysis

Statistical analyses were conducted using Excel 2018 and GraphPad Prism 6.0 software (La Jolla, Ca) with significance set at *P* < 0.05. Statistical evaluation utilized unpaired Student *t*-tests or analysis of variance (ANOVA) based on the characteristics of analysis. Data is expressed ± standard error of the mean.

## Results

### Mucus Samples From Acute Sinusitis Rabbits Support the Growth of PAO1 *in vitro*

**Figure 1:**
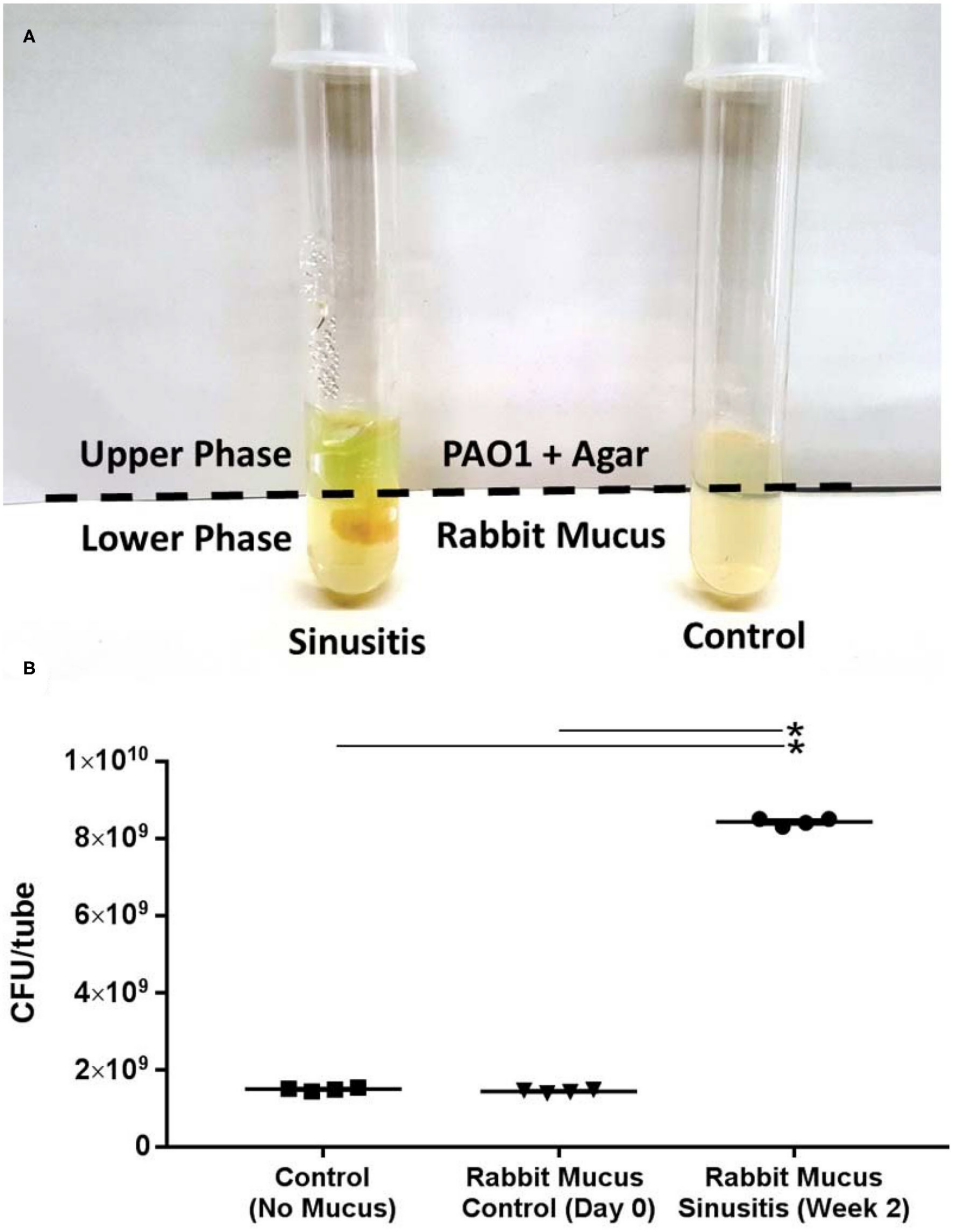
Mucus samples from Rabbit Sinusitis support the growth of PAO1 *in vitro*. **(A)** The mucus samples were co-cultured with PAO1 in an anaerobic minimal mucin medium. After 72 h, a diffusible blue-green pigment (pyocyanin) characteristic of *P*. *aeruginosa* growth was observed throughout the co-culture tubes containing sinusitis mucus (week 2). **(B)** Colony counts were significantly higher in those tubes contained with the mucus samples from week 2 (sinusitis: CFU/tube = 8.4 × 10^9^ ± 4.8 × 10^7^) compared to tubes containing control mucus (1.4 × 10^9^ ± 2.0 × 10^7^) or no mucus (1.5 × 10^9^ ± 2.1 × 10^7^), with a magnitude (5×) increase (*p* < 0.0001). *Represents statistical significance (*p* < 0.05).

To test whether the mucus samples from acute sinusitis in the rabbit model enriched with MDMs and SCFAs could simultaneously stimulate PAO1 growth, the mucus samples were co-cultured with PAO1 in a minimal mucin medium. Once the lower agar phase containing the mucus had solidified, PAO1 was suspended in buffered 0.7% agar medium without mucin (i.e., no carbon source) and was placed in the upper portion of the tube. This experimental setup establishes an oxygen gradient allowing anaerobes to grow, and restricts the movement of microbes but allows metabolites to freely diffuse (Flynn et al., 2016). Under these conditions, *P*. *aeruginosa* would be expected to achieve a higher cell density if provided with diffusible growth substrates from the mucus (lower phase). Co-culture growth was monitored over a 72-h period. After 72 h, a diffusible blue-green pigment (pyocyanin) characteristic of *P*. *aeruginosa* growth was observed throughout the co-culture tubes contained with sinusitis mucus (week 2) (Figure 1A). By contrast, no observable pigment was produced in the tubes contained with control mucus (Day 0) or without any mucus. Colony counts of the upper phase were significantly higher in those tubes containing mucus samples from week 2 (sinusitis: CFU/tube = 8.4 × 10^9^ ± 4.8 × 10^7^, *n* = 4) compared to tubes containing control mucus (1.4 × 10^9^ ± 2.0 × 10^7^, *n* = 4) or no mucus (1.5 × 10^9^ ± 2.1 × 10^7^, *n* = 4), with a magnitude 5-fold increase (*p* < 0.0001) (Figure 1B).

### Mucus Samples From Acute Sinusitis Rabbits Contain Short Chain Fatty Acids (SCFA)

**Figure 2:**
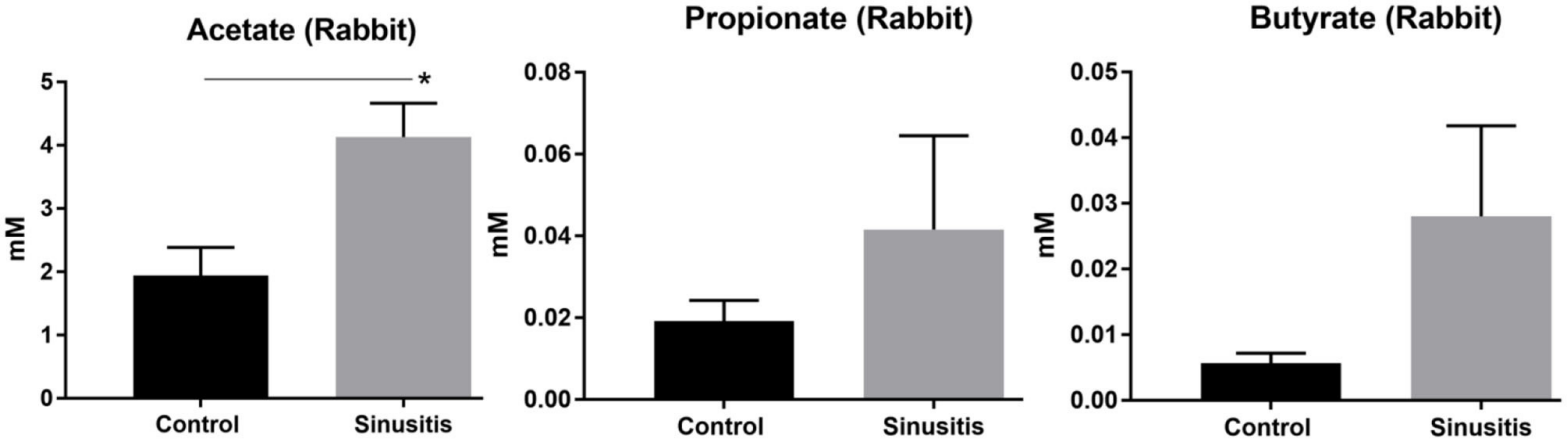
Concentrations of short chain fatty acids (SCFA) in mucus samples from rabbits. Acetate concentrations were significantly higher in the mucus samples collected on week 2 (Sinusitis), relative to day 0 (Control) (4.13 ± 0.53 vs. 1.94 ± 0.44 mM, *p* < 0.01, *n* = 4). Propionate and butyrate concentrations were higher in the mucus samples from sinusitis compared to those from control (propionate = 0.042 ± 0.023 vs. 0.019 ± 0.005 mM, *p* = 0.38; Butyrate = 0.028 ± 0.014 vs. 0.005 ± 0.002 mM, *p* = 0.085) but statistical significance was lacking (*n* = 4). All groups consisted of at least four experiments. *Represents statistical significance (*p* < 0.05).

To provide evidence of fermentative activity *in vivo*, 3 SCFAs (acetate, propionate and butyrate) were quantified using GC-MS within the mucus samples from rabbits on day 0 (Control) and week 2 (Sinusitis). SCFAs were found at millimolar (or less) concentrations in all mucus samples and we were able to detect all three SCFAs (Figure 2). Acetate concentrations were significantly higher in the mucus samples collected on week 2 (Sinusitis), relative to day 0 (Control) (4.13 ± 0.53 vs. 1.94 ± 0.44 mM, *p* < 0.01, *n* = 4). Propionate and butyrate concentrations were not significantly elevated in the mucus samples from sinusitis compared to those from control [Propionate = 0.042 ± 0.023 vs. 0.019 ± 0.005 mM, *p* = 0.38 (*n* = 4); Butyrate = 0.028 ± 0.014 vs. 0.005 ± 0.002 mM, *p* = 0.085 (*n* = 4)] even though there was a trend.

### Human Mucus Samples From CRS With *P. aeruginosa* Contain Significantly Higher SCFAs

**Figure 3:**
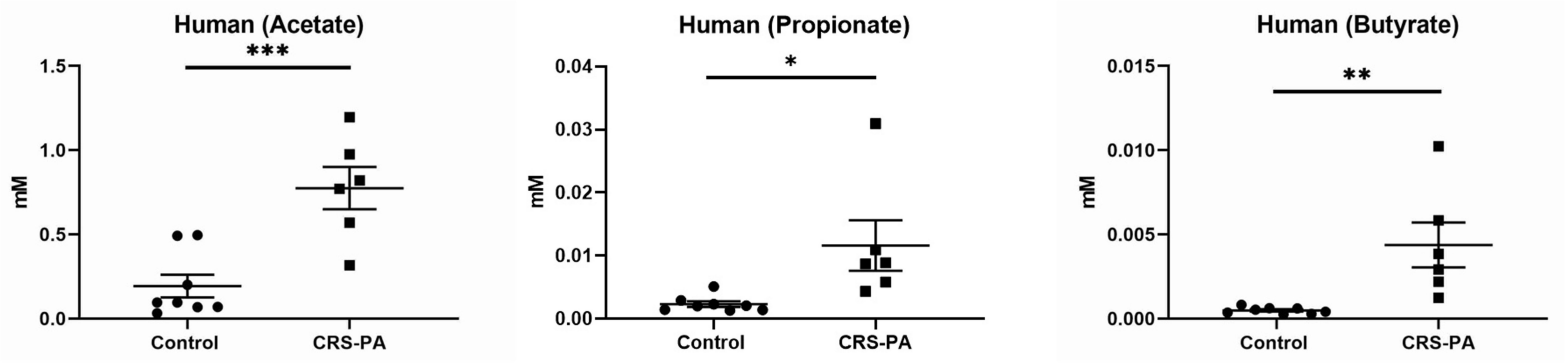
Concentrations of short chain fatty acids (SCFA) in human mucus samples SCFAs from CRS patients with *P. aeruginosa* (PA) (*n* = 6) were compared to those from controls (*n* = 8): (1) acetate = 0.77 ± 0.13 vs. 0.19 ± 0.07 mM (*p* < 0.001; (2) propionate = 0.012 ± 0.004 vs. 0.002 ± 0.000 (*p* = 0.019; (3) butyrate = 0.004 ± 0.001 vs. 0.0005 ± 0.000 (*p* < 0.01) [*represents statistical significance *p* < 0.05, **p < 0.01, and ***p < 0.001].

**Figure 4:**
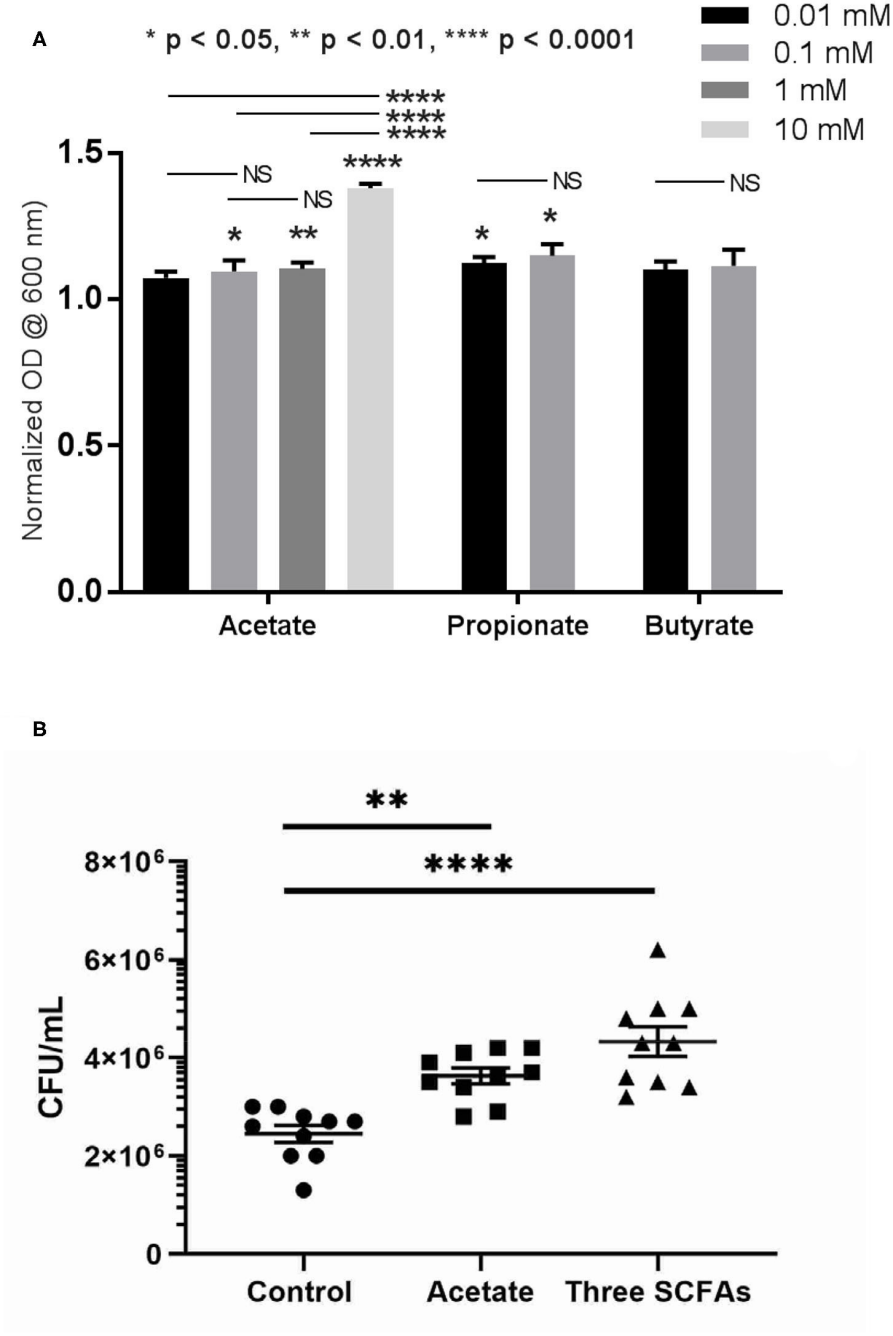
Growth of PAO1 with SCFAs. **(A)** PAO1 growths were compared to controls in the presence of different concentrations of acetate (between 0.1 and 10 mM): normalizzed OD values at 600 nm: 0.01 mM = 7.3 ± 0.02 (*n* = 4), 0.1 mM = 9.6 ± 0.04 (*n* = 4), 1 mM = 11.04 ± 0.02 (*n* = 4), and 10 mM 13.8 ± 0.02 (*n* = 4). In the presence of propionate, statistical significance was noted in both concentrations compared to controls (Normalized OD values at 600 nm: 0.01 mM = 12.3 ± 0.02 (*n* = 4), 0.1 mM = 15 ± 0.04 (*n* = 4). However, no statistically significant growth of PAO1 was seen with butyrate at these two concentrations [0.01 mM = 10 ± 0.03 (*n* = 4), 0.1 mM = 11.5 ± 0.05 (*n* = 4), *p* = 0.096]. (one-way ANOVA, NS = no significance). **(B)** Colony counts were significantly higher when PAO1 strains were grown with acetate alone or three SCFAs compared to controls (Control = 2.45 × 10^6^ ± 1.7 × 10^5^ CFU/ml (*n* = 10), Acetate = 3.63 × 10^6^ ± 1.58 × 10^5^ CFU/ml (*n* = 10), 3 SCFAs = 4.33 × 10^6^ ± 3.0 × 10^5^ CFU/ml (*n* = 10), ANOVA, *p* < 0.0001, (*p < 0.05, **p < 0.01, and ****p < 0.0001).

To provide the evidence of fermentative activity in human CRS with *P. aeruginosa*, we analyzed the presence of SCFAs in human mucus samples from controls and CRS with *P. aeruginosa*. Of those human mucus samples collected in the clinic or OR, eight samples were collected from controls and six from CRS with *P. aeruginosa*. All CRS patients with *P. aeruginosa* presented with purulence in the sinus cavity (without any recent use of oral or intravenous antibiotics for at least 2 weeks). Of those 6 CRS with *P. aeruginosa* mucus samples, mucus-degrading microbes were present in five samples (83.3%) from our hospital clinical microbiology laboratory report (Table 1). Using GC-MS, 3 SCFAs (acetate, propionate, and butyrate) were quantified in human mucus samples collected from eight controls and 6 CRS patients with *P. aeruginosa* with active purulent drainage. SCFAs were found at mM concentrations in all mucus samples (**Figure 4**). SCFAs from CRS patients with *P. aeruginosa* were also significantly higher than those from controls: (1) acetate = 0.77 ± 0.13 vs. 0.19 ± 0.07 mM (*p* < 0.001), (2) propionate = 0.012 ± 0.004 vs. 0.002 ± 0.000 (*p* = 0.019), (3) butyrate = 0.004 ± 0.001 vs. 0.0005 ± 0.000 (*p* < 0.01) (Figure 3). Collectively, data presented here demonstrate the presence of significantly higher quantities of fermentation metabolites in CRS with *P. aeruginosa*.

**Table 1:** Presence of Mucin degrading microbes in *P. aeruginosa* culture positive patients.

**No**.	**Age**	**Comorbidities**	**Location of culture**	**Mucin degrading microbes**
#1	69	DM	Frontal sinus	*Streptococcus intermedius*
#2	39	Asthma, Mannose-binding lectin protein deficiency	Ethmoid sinus	*Enterobacter cloacae*
#3	32	CF (ΔF508/ΔF508)	Frontal sinus	*Cutibacterium acnes, Enterobacter cloacae*
#4	71	Immunocompromised (post-KT)	Maxillary sinus	None
#5	78	DM, Prostate cancer	Middle meatus	*Klebsiella oxytoca*
#6	52	DM, COPD	Ethmoid sinus	*Rothia mucilaginosa, Streptococcus salivarius*

*DM, Diabetes mellitus; CF, Cystic fibrosis; KT, Kidney transplantation; COPD, Chronic obstructive pulmonary disease*.

### SCFAs Increase PAO1 Growth *in vitro*

To understand the role of SCFAs in the growth of PAO1 *in vitro*, the growth of PAO1 with multiple concentrations of SCFAs was monitored. Concentrations of SCFAs were chosen based on the previous HPLC findings above (Figure 2). As acetate concentrations were above 1 mM in the rabbit mucus samples, we utilized four different concentrations of acetate (0.01, 0.1, 1, and 10 mM). Because propionate and butyrate were <1 mM, two different concentrations (0.01 and 0.1 mM) below 1 mM were used. Normalized OD values were compared to the average OD from control (without adding SCFAs) for acetate, propionate, and butyrate (Figure 4A). In the presence of acetate, PAO1 exhibited increased growth from 0.01 to 10 mM and statistical significance was noted between 0.1 and 10 mM compared to control [normalized OD values at 600 nm: 0.01 mM = 7.3 ± 0.02 (*n* = 4), 0.1 mM = 9.6 ± 0.04 (*n* = 4), 1 mM = 11.04 ± 0.02 (*n* = 4), and 10 mM 13.8 ± 0.02 (*n* = 4), *p* < 0.0001 (ANOVA with *post-hoc* Tukey–Kramer)]. In the presence of propionate, statistical significance was noted in both concentrations [normalized OD values at 600 nm: 0.01 mM = 12.3 ± 0.02 (*n* = 4), 0.1 mM = 15 ± 0.04 (*n* = 4), *p* < 0.01 (ANOVA)]. However, the growth increases of PAO1 observed with butyrate were not statistically significant in the analysis of these two concentrations [0.01 mM = 10 ± 0.03 (*n* = 4), 0.1 mM = 11.5 ± 0.05 (*n* = 4), *p* = 0.096 (ANOVA)]. Additionally, as acetate is the major SCFA (at least 50 or 200-fold higher than propionate or butyrate) in Figure 3, the growth of PAO1 strains was compared in the presence of acetate alone (0.5 mM) vs. 3 SCFAs (acetate 0.5 mM, propionate 0.01 mM, Butyrate 0.005 mM). Colony counts were significantly higher when PAO1 strains were grown with acetate alone or 3 SCFAs compared to controls (Control = 2.45 × 10^6^ ± 1.7 × 10^5^ CFU/ml (*n* = 10), Acetate = 3.63 × 10^6^ ± 1.58 × 10^5^ CFU/ml (*n* = 10), Three SCFAs = 4.33 × 10^6^ ± 3.0 × 10^5^ CFU/ml (*n* = 10), ANOVA, *p* < 0.0001) (Figure 4B). Even though there was a trend toward higher colony counts when PAO1s were treated with 3 SCFAs, statistical significance was lacking between the acetate alone and 3 SCFAs (Tukey's multiple comparison test, *p* = 0.077).

### Targeting Anaerobes Halts the Radiographic Evidence of Sinusitis Progression

**Figure 5:**
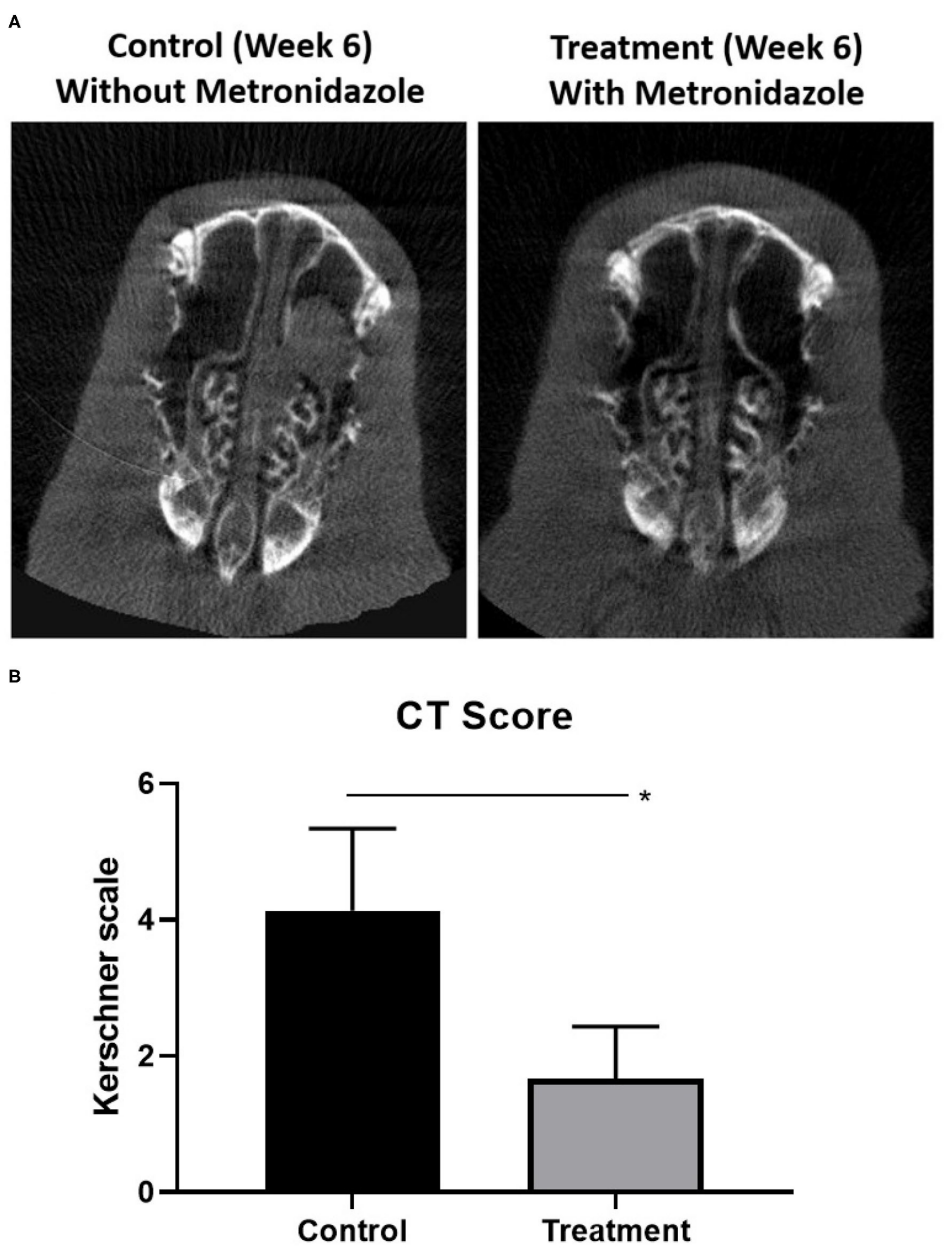
Radiographic progression of sinusitis when targeting anaerobes. **(A)** At week 6 (midpoint between acute and chronic), the metronidazole treatment group (*n* = 3) had marked improvement of opacification compared to the control group without treatment (*n* = 3). **(B)** Scores were significantly higher in rabbits without metronidazole treatment (Kerschner scale, *n* = 3, 4.13 ± 1.21) compared to those rabbits treated with metronidazole (*n* = 3, 1.67 ± 0.76) (*p* = 0.040) at week 6 (*p < 0.05).

To evaluate whether targeting fermentative anaerobes halts sinusitis progression from acute to chronic stages, acute sinusitis rabbits (week 2) were treated with metronidazole for 5 days. At week 6 (midpoint between acute and chronic), the metronidazole treatment group (*n* = 3) had marked improvement of opacification compared to the control group without treatment (*n* = 3) (Figure 5). CT scores were significantly higher in rabbits without metronidazole treatment (Kerschner scale, *n* = 3, 4.13 ± 1.21) compared to those rabbits treated with metronidazole (*n* = 3, 1.67 ± 0.76) (*p* = 0.040) at week 6. Radiographically, the progression to sinusitis was halted by metronidazole treatment.

## Discussion

In this study, we investigated the role of SCFAs (produced by mucin fermenting anaerobes) in the growth of *P*. *aeruginosa* in rhinosinusitis. To our knowledge, this is the first study to quantitatively assess the presence of SCFAs in the human mucus including patients with CRS. While *Pseudomonas* ineffectually utilizes mucins as a carbon source on its own (Flynn et al., 2016), we determined that mucin fermentation by MDMs can stimulate the growth of *P*. *aeruginosa*. Moreover, we revealed that SCFAs were also abundant and available in CRS patients with and without *P*. *aeruginosa*. Together, these results suggest that the high levels of utilizable metabolites present in sinus mucus may be derived from bacterial mucin degradation by anaerobes in the sinus cavity, which may contribute to the establishment and progression of recalcitrant CRS (Cho et al., 2020).

Under normal oxygen conditions, most bacteria preferentially oxidize glucose and other saccharides to pyruvate and shuttle pyruvate through the citric acid cycle. Both processes require oxygen as the final electron acceptor. In anaerobic conditions, bacteria must choose an alternative route by using the energy of another biochemical reaction, and thus bypassing oxidative respiration (Zumft, 1997; Ragsdale and Pierce, 2008). The process of fermentation involves the release of energy from compounds without utilizing exogenous oxygen (e.g., muscle cells during exercise through the formation of lactic acid; Ghorbani et al., 2015). Cystic fibrosis (CF) airway disease is one condition which gives rise to persistent bacterial colonization, coupled with an anaerobic microenvironment. The CF airway includes a thick mucus layer, rendered hypoxic through the metabolism of host immune cells and bacteria. Under these circumstances, bacteria can produce SCFAs through the fermentation process. Fermentation of carbohydrates within the mucus results in the formation of SCFAs (e.g., propionate, butyrate, and acetate), which can thus influence the progression and resolution of infection and inflammation in the airways (Ghorbani et al., 2015). SCFAs have been shown to downregulate immune cell inflammatory responses, promote neutrophil chemotaxis, induce inflammatory protein expression in epithelial cells, inhibit proliferation, and strengthen epithelial tight junctions in the gastrointestinal tract (Ferreira et al., 2012; Vieira et al., 2012). In the large intestine, SCFAs are found at concentrations ranging from 30 to 150 mM, which is significantly higher than the concentrations found in the airway (Mortensen and Clausen, 1996; Smith et al., 2013). While high mM concentrations impair growth, low mM concentrations (<10 mM) boost the growth of potential pathogens (e.g., *Pseudomonas*) and upregulate IL-8 in CF primary airway epithelium (Ghorbani et al., 2015). These molecules easily penetrate the airway tissue because of their low molecular weight and subsequently interrupt host cell activity and host defense systems by inducing apoptosis in fibroblasts and lymphocytes (Tonetti et al., 1987; Kurita-Ochiai et al., 1995; Sato et al., 2016). It is also possible that SCFAs may influence *P. aeruginosa* biofilm formation, which will be investigated in future experiments.

It is interesting to note that SCFA levels were higher in rabbits than human samples in both controls and non-controls. Every species appear to have different microbial fermentation patterns even if they have similar diets (Kroliczewska et al., 2018). This is the first report (to our knowledge) to evaluate SCFA levels in rabbit sinonasal mucus and we will require more numbers to strictly define normal SCFA levels in rabbit sinuses. However, the high fiber intake in the diet encourages the growth of species that ferment fiber into metabolites as SCFAs, and thus rabbits' baseline could be higher than humans as previously shown in the gut (Cummings et al., 1987; Tomova et al., 2019). Furthermore, for experimental conditions, we intentionally created an anaerobic environment in the sinus cavity and the SCFA levels were measured during the acute sinusitis phase in the rabbit model. By contrast, the human samples were collected from the (postoperative) sinuses or middle meatus in CRS patients. Therefore, the SCFAs from rabbits may have been generated at higher levels than observed in human samples.

Based on our experiments, all 3 SCFAs were significantly higher in CRS with *P. aeruginosa*. Our clinical results are also consistent with CF bronchoalveolar lavage fluid findings in other studies (Ghorbani et al., 2015; Mirkovic et al., 2015; Flynn et al., 2016). The ratio of propionate to acetate (1:50) in our human sinusitis samples was comparable to Flynn et al.'s experiments in human CF sputum/saliva samples (Flynn et al., 2016). As explained by Flynn et al., only a small amount of propionate is generated *in vivo* or it may be used by microorganisms in a cross-feeding relationship. When we compared the CFUs between the acetate alone and three SCFAs, an additional growth of *P. aeruginosa* could be clinically presumable in the presence of multiple nutrients, even though statistical significance was lacking in this experiment. When comparing the total SCFA concentrations before and after antibiotic treatment in patients presenting with a CF pulmonary exacerbation, the mean SCFA concentrations were significantly lower after the treatment (Ghorbani et al., 2015).

**Figure 6:**
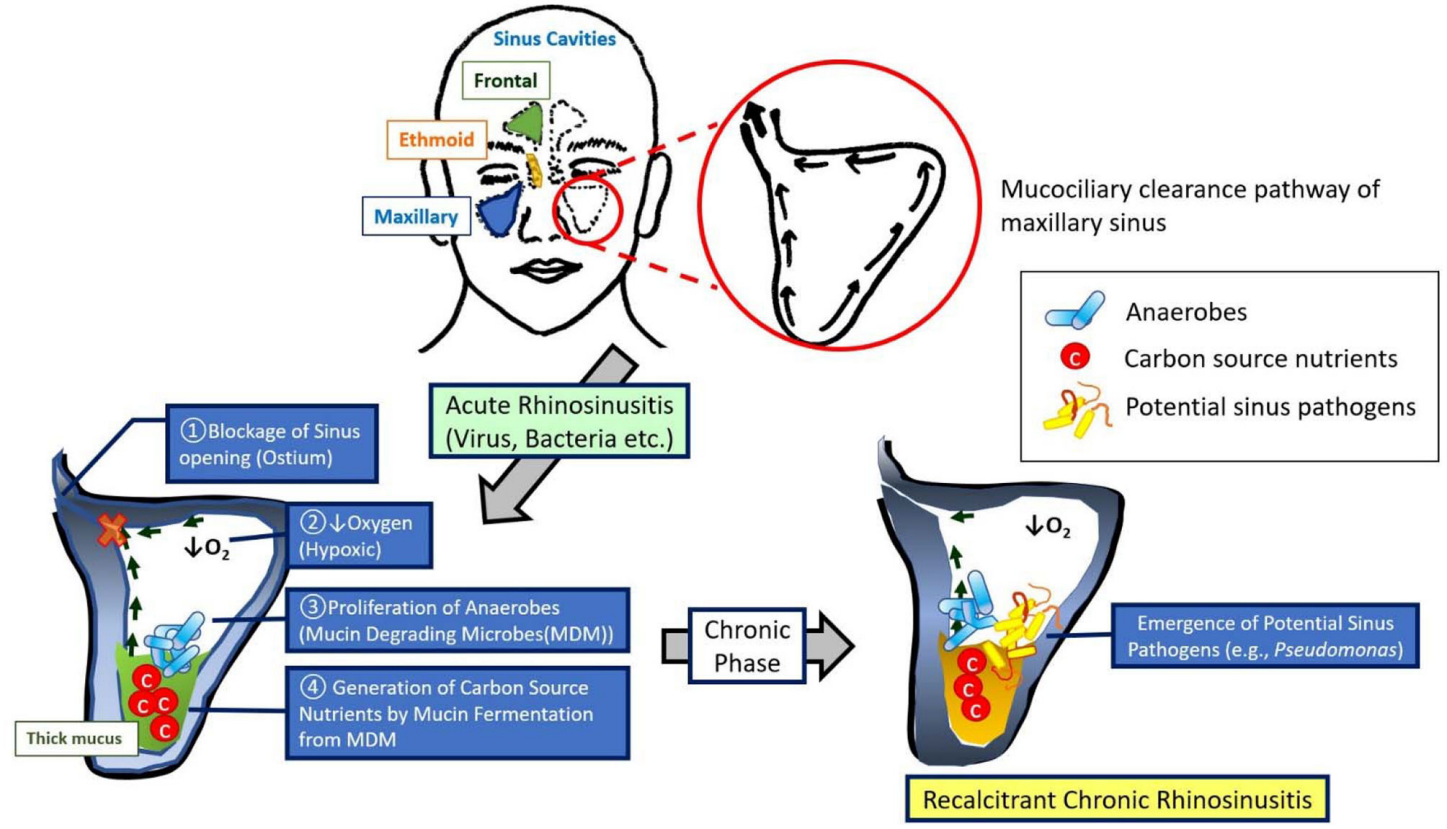
Model for the development of recalcitrant chronic rhinosinusitis. This model demonstrates a sequence of events that augment our understanding of recalcitrant CRS pathophysiology: (1) blockage of sinus ostia generates a shift in microbiota to a predominance of anaerobes, mucin metabolites [short chain fatty acids (SCFAs)] produced by these anaerobes are abundant within the mucus during an acute anaerobic stage, and (3) the shift from acute to chronic sinus inflammation is associated with a robust increase in pathogenic bacteria (e.g., *P. aeruginosa*).

Our results suggest the role of hypoxia in recalcitrant CRS pathophysiology (Figure 6). Hypoxia due to sinus closure at the ostiomeatal complex at the junction of the ethmoid and maxillary sinuses is widely considered a major pathogenic mechanism leading to the development of CRS and is strongly supported by proof that hypoxia is present at the surface epithelium in diseased sinuses of closed ostia (Aanaes et al., 2011). Targeting mucin-fermenting anaerobes and their metabolites could be a novel therapeutic strategy for the treatment of CRS by restoring the microbial community in diseased sinuses to the original non-infected pristine status.

There are several limitations to this study. Our hypothesis is from the animal model not human subjects, and this may not reflect the human *in vivo* environment. Even though some well-controlled microbiota studies using animal models have also shown inter-study variations due to confounding factors (e.g., origin, maternal effects, environmental conditions) (Nguyen et al., 2015), the advantages of the rabbit model are numerous in that it enables us to control for a number of variables that are inherent in human samples including genetics, medical history, environmental allergies/pollutants, and medication use (Cho et al., 2018). As this is a clinical pilot study to assess the concentrations of SCFAs in CRS patients, the size and homogeneity of the clinical samples limit the study's generalizability. In addition, we did not include any microbiome-related analyses or molecular based quantification of the microorganisms that colonize the mucus layers of human samples. A much larger group of patients will be recruited for future studies. Additionally, *Bacteroides* (one of the most common anaerobes in the rabbit model) is not one of the common anaerobes in human sinusitis and there could be other nutrients other than SCFAs, which are derived by fermentation that sustain bacterial growth in humans. Therefore, we are planning to test the capability of patient-derived, anaerobic microbiota (Taxa: *Streptococcus, Peptostreptococcus, Propionibacterium, Fusobacterium*, and *Prevotella*) to support the growth of *P. aeruginosa* isolates using an *in vitro* and *in vivo* cross-feeding co-culture model.

## Conclusion

Given that SCFAs are solely derived from bacterial fermentation, our experiments propose a critical role for mucin-fermenting bacteria in generating carbon-source nutrients for pathogenic bacteria in the airway. Mucin fermenting anaerobes may contribute to the development of recalcitrant CRS by degrading mucins, thus providing nutrients for potential pathogens like *P. aeruginosa*.

## Data Availability Statement

The datasets generated for this study are available on request to the corresponding author.

## Ethics Statement

The studies involving human participants were reviewed and approved by Institutional Review Board (IRB approval number 300003118) at the University of Alabama at Birmingham. The patients/participants provided their written informed consent to participate in this study. The animal study was reviewed and approved by Institutional Animal Care and Use Committee (IACUC Approval number 21687) at the University of Alabama at Birmingham (UAB).

## Author Contributions

D-YC designed the study, carried out the experiments, and took the lead in writing the manuscript with support from RH, SR, WS, and BW (concept of experiments and editing). DS, DL, HT, CRW, and SZ carried out the rabbit and *in vitro* experiments and contributed to sample preparation. JG performed statistical analysis. All authors provided critical feedback, helped shape the research, analysis, and manuscript.

## Conflict of Interest

The authors declare that the research was conducted in the absence of any commercial or financial relationships that could be construed as a potential conflict of interest.
